# Association of Relative Telomere Length and LINE-1 Methylation with Autism but not with Severity

**DOI:** 10.1007/s10803-023-05965-0

**Published:** 2023-04-04

**Authors:** Sohair Salem, Engy Ashaat

**Affiliations:** 1 Molecular Genetics & Enzymology Department, Human Genetics and Genome Research Institute, National Research Centre, Cairo, Egypt; 2 Clinical Genetics Department, Human Genetics and Genome Research Institute, National Research Centre, Cairo, Egypt

**Keywords:** Autism, Relative Telomere Length, LINE-1, Methylation

## Abstract

Autism is associated with genomic instability, which is regulated by telomere length (TL) and index of global methylation (LINE-1). This study will determine relative TL (RTL) and LINE-1 methylation percentage for 69 patients and 33 control subjects to evaluate their potential role as biomarkers for autism. The results displayed a significant decrease of both RTL and LINE-1 methylation in autistic cases relative to controls (*P* < 0.001). Analysis of receiver operating characteristics curve revealed that both of RTL and LINE-1 methylation percentage have the ability to serve as autism biomarkers (area under the curve = 0.817 and 0.889, respectively). The statistical analysis revealed positive correlation between the two biomarkers (correlation coefficient = 0.439 and *P* < 0.001)*.*

## Introduction

Autism spectrum disorder (ASD) is a group of early-onset and lifelong neurodevelopmental disorders with distinct phenotype of behavioural characteristics (ranging from mild to severe), including impairments in social communication and restricted repetitive behaviours, interests, or activities. Co-occurrence with other disorders, such as motor delays, depression, intellectual disability, anxiety disorder, gastrointestinal disturbances, attention deficit hyperactivity disorder, and seizure disorder, is a common event in ASD patients (Fyke & Velinov, 2021). ASD affects one in every 54 children aged 8 years and is 4.3 times more prevalent in boys than in girls (Maenner et al., 2020). A recent study in Egypt determined the prevalence of ASD to be 5.4/1000 (Yousef et al., 2021).

Complexity and risk factors heterogeneity, including environmental and genetic factors of ASD have been well documented. For example, although numerous single genes, copy-number, and double hit models have been identified in autism, approximately 70% of autistic subjects have unknown causes (Homs et al., 2016). In addition, epigenetic factors such as DNA methylation (Kimura et al., 2019), histone modifications (Sun et al., 2016), and non-coding RNAs (Tonacci et al., 2019) have been implicated in ASD incidence.

A telomere is an evolutionarily conserved tract of repetitive DNA sequence located at the end of a chromosome and bound by a series of specialized proteins to function as protective cap for the chromosome. Mammalian telomeric DNA comprises ~ 5–15 kb of double-stranded tandem repeats (5ʹTTAGGG repeats for the G-rich strand and 5ʹCCCTAA repeats for the complementary C-rich strand) ended in a 3’ single-stranded overhang of ~ 12–300 bases. During cell division, the human telomere loses approximately 50–200 base pairs (Stewart et al., 2012); consequently, telomere length (TL) is a hallmark of cellular senescence and organismal aging, and its shortening is a biomarker for numerous diseases (Zhao et al., 2009).

One of best-characterized epigenetic mechanisms is cytosine residues methylation at CpG sites. 60–90% of CpGs throughout the genome were found to be methylated (Nguyen et al., 2010). Long interspersed element-1 (LINE-1) is a ~ 6,000 bp-long transposable mobile element that exists in over 500,000 copies to represent approximately 17% of the human genome. LINE-1 contains a 5ʹ untranslated region (UTR) with an internal promoter, two non-overlapping open reading frames (ORF1 and ORF2), and a 3ʹ UTR (Tangsuwansri et al., 2018). The proteins encoded by LINE-1 (ORF1p and ORF2p) are responsible for its retrotransposition after their assembly into a functional ribonucleoprotein (RNP) particle. LINE-1 contributes to genomic instability and altered gene expression via a “copy-and-paste” mechanism of amplification (Belancio, 2015). DNA methylation and histone modifications are suggested to play a role in regulating transcription and retrotransposition of LINE-1. Hypomethylation of LINE-1 leads to its reverse transcription into DNA sequence and transposition throughout the genome, contributing to gene disruption and genomic instability (Swets et al., 2016). Altered methylation pattern of LINE-1 is associated with numerous diseases, including cancer (Swets et al., 2016), heart diseases (Guarrera et al., 2015), and neurodevelopmental disorders (Shpyleva et al., 2018). Due to the extensive genomic distribution of LINE-1 and its high methylation level in most normal tissues, LINE-1 methylation regarded as an index and surrogate marker for global DNA methylation (Guarrera et al., 2015).

Both TL and LINE-1 are involved in regulating genomic stability, which is implicated in autism (Williams et al., 2013); therefore, this study will evaluate relative TL (RTL) and LINE-1 methylation as biomarkers for autism, as well as their correlation to each other, disease severity, and other clinical characteristics.

## Subjects and Methods

### Subjects

The research was conducted in 2019–2020 with 69 Egyptian participants (55 males and 14 females) who met the DSM-V diagnostic criteria for ASD. Diagnosis and severity rating of autism were carried out by Childhood Autism Rating Scale (CARS). Any syndromic ASD was ruled out. The ages of patients ranged from 2 to 20 years (mean age: 6.8 years) and they are classified as children (2–10 year) and adolescents (11–20 year). The ages of the subjects in each group are distributed in small rang, except for three cases that were considered as outliers. All patients were recruited from the outpatient clinic of Clinical Genetics Department, Centre of Excellence of Human Genetics, National Research Centre (NRC), Egypt. They were subjected to detailed medical history assessment (personal, pregnancy, delivery, perinatal and neonatal). Subsequently, 33 age-matched healthy subjects were analyzed as the control group. The onset of symptoms, their progress, the presence of a sleep disorder and gastrointestinal (GIT) manifestation were meticulously documented. EEG was performed on all patients at the Neurophysiological unit, Faculty of Medicine, Kaser El-Ainy, Cairo University (patient’s characteristics are listed in Table 1). It was done under sedation by chloral hydrate for 2 h. Patients provided informed and written consent to participate and the study protocol was approved by the ethical research committee of the NRC (Registration number: 19033).

**Table 1:** Demographic and clinical data of autistic subjects

Data	Number (%)
Sex	
Males	55 (79.7)
Females	14 (20.3)
Age class	
Children	56 (81.1)
Adolescents	13 (18.9)
Severity	
Mild	34 (49.3)
Moderate	22 (31.8)
Severe	13 (18.9)
Consanguinity	
Positive	19 (27.5)
Negative	50 (72.5)
EEG abnormalities	
Positive	28 (40.6)
Negative	41 (59.4)
GIT abnormalities	
Positive	44 (63.8)
Negative	25 (36.2)
Sleep disorder	
Positive	11 (16)
Negative	58 (84)

*GIT* Gastrointestinal

### Telomere Length Measurement

DNA was extracted from peripheral blood leukocytes using standard salting out protocol. *36B4* was used as reference single-copy gene. 80 n of extracted DNA was subjected to qPCR using HERA SYBR green master mix (Willowfort, UK) and primers for telomere (Ujvari et al., 2012) and 36B4 (Willeit et al., 2014) (Primers sequences are listed in Table 2). Reaction was incubated for 2 min at 95 °C and then amplified over 40 cycles of 10 s at 95 °C and 30 s at 60 °C (for telomeres) or 56 °C (for *36B4*). The Reactions were run in light cycler 480 II (Roch). For quality control, A no-template control and duplicate calibrator samples were used in all runs to allow for a comparison of the results across all runs (Svikle et al., 2022). RTL was reflected by T/S values (telomere/*36B4* ratio): T/S = 2^−∆^Ct (∆Ct = Ct telomere − Ct*36B4*).

**Table 2:** Sequence of primers used in qPCR and MSP

Name	Sequence 5 → 3	Refs.
Telomere F	GGTTTTTGAGGGTGAGGGTGAGGGTGAGGGTGAGGGT	Ujvari et al. (2012)
Telomer R	TCCCGACTATCCCTATCCCTATCCC TATCCCTATCCCTA	
36B4 F	CAGCAAGTGGGAAGGTGTAATCC	Willeit et al. (2014)
36B4 R	CCCATTCTATCATCAACGGGTACAA	
LINE-1 M-F	GAGGTATTGTTTTATTTGGGAAGC	
LINE-1 M-R	TACTAACAATCAACGAAATTCCGTA	
LINE-1 U-F	AGGTATTGTTTTATTTGGGAAGTGT	
LINE-1 U-R	TACTAACAATCAACAAAATTCCATA	

*F* Forward, *R* Reverse, *M* Methylated, *U* Un-methylated

### LINE-1 Methylation Analysis

The extracted DNA was subjected to bisulfite modification using the EpiTect Bisulfite kit (Qiagen). The Initial starter amount of DNA was 1.3 µg and the final elution volume was 40 µl. Bisulfite-treated DNA was subjected to methylation specific PCR (MSP), in which two distinct sets of primers (methylated and unmethylated) were employed and amplified. Each MSP reaction was conducted in 15 µl reaction using 7.5 µl of maxima SYBR green master mix (Thermo Scientific), 2 µl of treated DNA, and 150 nM of each primer. The percentage of methylation was calculated using the CT method follows: % meth = 100/[1 + 2^ΔCt (meth. − un−meth.)^]%. The LINE-1 sequence was downloaded from NCBI (https://www.ncbi.nlm.nih.gov/) (L19088.1). Primers were designed using the free online methprimer tool (http://www.urogene.org/methprimer) which identified two CpG islands with 371 and 101 bp in length, and the primers were designed to span 209 and 210 bp of the first island. The number of CpG sites covered by each primer are zero for methylated forward, two for methylated reverse, one for unmethylated forward and two for unmethylated reverse (sequences are listed in Table 2).

### Statistical Analysis

The statistical analysis was carried out using IBM SPSS statistics (Statistical Package for Social Sciences) software version 18.0, IBM Corp., Chicago, USA, 2009. Descriptive statistics were done for quantitative data as minimum and maximum of the range as well as mean ± SD. A Modified Z-score test was used to detect outliers of age. Difference of RTL and LINE-1 methylation percentage between different groups (controls and autistic subjects, males and females, children and adolescents and positive and negative consanguinity) was determined by independent T test. We applied univariate linear model with gender and age as covariates to adjust sex and age effect. In this model, age is reclassified into four groups with corresponding scores (score1, 2–5 years; score 2, 5.1–10 years; score 3, 10.1–15 years; and score 4, 15.1–20 years). Also, sex was scored as a categorical variable as 1 (males) or 2 (females). ANOVA was utilized to compare RTL and LINE-1 methylation levels between different severity groups (mild, moderate, and severe). To determine the effectiveness of RTL and LINE-1 methylation as a discriminatory biomarker for autistic patients, the receiver operating characteristic (ROC) curve was analyzed. Next, Binary logistic regression was used to analyze the combined ROC curve of both biomarkers. The correlation between the two biomarkers was calculated using Pearson’s correlation coefficient. *Spearman’s correlation coefficient was employed to determine the degree of correlation between* RTL or LINE-1 methylation level and other characteristics, such as age, sex, consanguinity, severity, EEG, sleep disorders, and gastrointestinal disturbance. The level of significance was set at *P* value < 0.05.

## Results

### RTL as a Biomarker for Autism

**Fig. 1:**
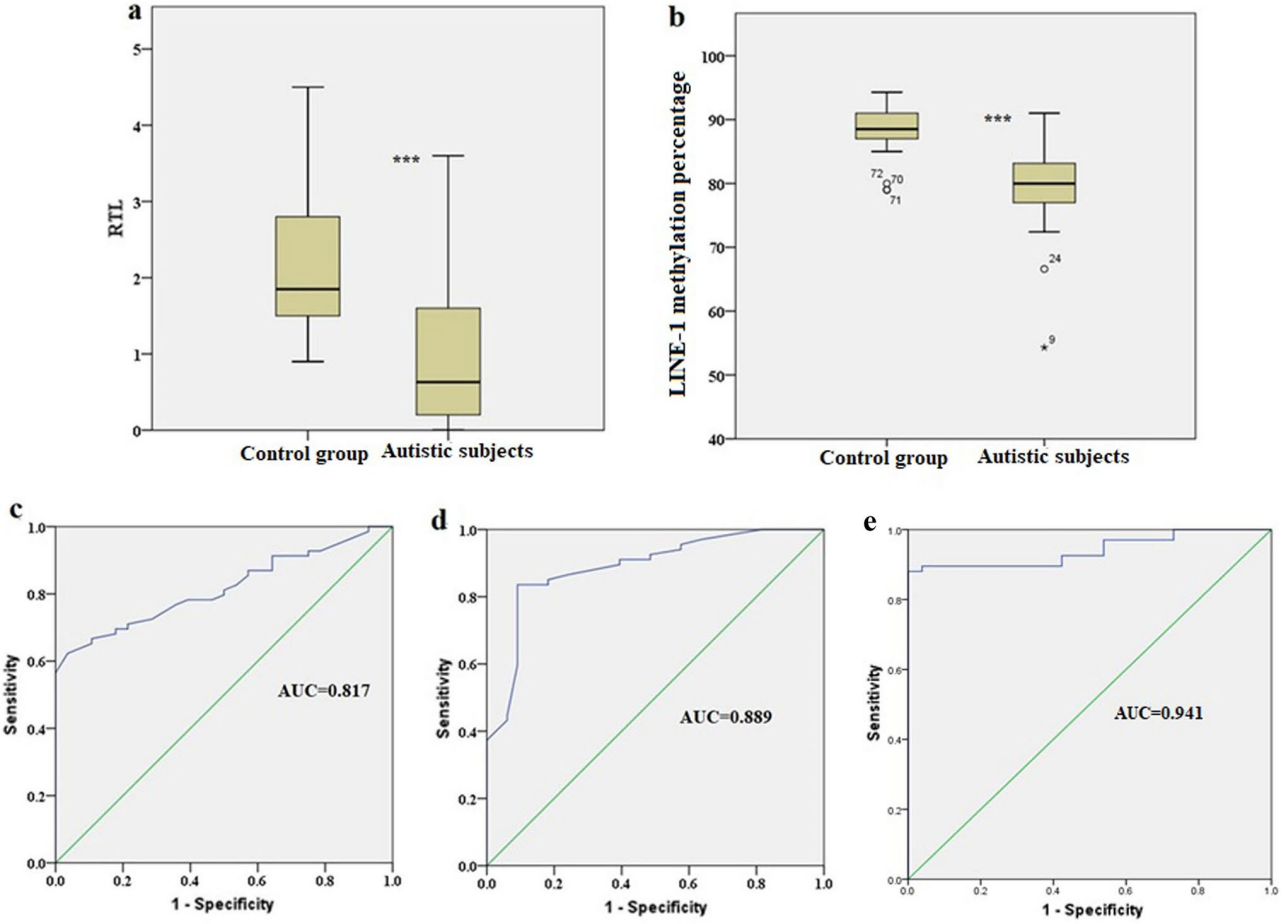
Altered RTL and LINE-1 methylation level in autistic patients. **a** and **b** represent boxplot for distribution of RTL (**a**) and LINE-1 methylation percentage (**b**) in autistic and control groups. **c**, **d** and **e** showing ROC curve analysis of RTL (**c**), LINE-1 methylation percentage (**d**) and the combined biomarkers (**e**) for autism prediction

RTL is significantly shorter in autistic patients (1, ± 0.97) compared to the group (2.1; ± 0.93), *P* < 0.001. We have applied ROC curve analysis to test the predictive ability of RTL to distinguish autistic individuals from normal subjects based on the value of area under the curve (AUC), which is considered more discriminative at more higher value (maximum value is 1 and minimum value is 0.5) (Hajian-Tilaki, 2013). Based on our data, RTL is considered a predictive biomarker for autism; AUC = 0.817 and *P* < 0.001 (Table 3 and Fig. 1). By comparing RTL between children and adolescents, no statistically significant difference was found (*P* = 0.880). However, the mean of RTL increased in cases with positive consanguinity (1.3; ± 1) than in cases with negative consanguinity (0.78; ± 0.7) (*P* = 0.058), and the Spearman’s correlation coefficient between RTL and consanguinity was not statistically significant: r = 0.249 and *P* = 0.053. Similarly, there was no significant association between RTL and sex: r = 0.228 and *P* = 0.074 (Table 4).

**Table 3:** ROC curve analysis and distribution of RTL and LINE-1methylation percentage between autistic and control subjects

Biomarker	ROC curve		Independent T test				Adjusted *P* value*
	AUC	*P* value	Mean (± SD)	*P* value	95% CI		
					Lower	Upper	
RTL							
Controls (N = 28)	0.817	*P* < .001	2.1 (0.93)	*P* < .001	− 1.58869	− .73010	0.005
Cases (N = 69)			1 (0.97)				
LINE-1 methylation percentage							
Controls (N = 33)	0.889	*P* < .001	88.2 (3.8)	*P* < .001	− 10.61465	− 6.12981	0.005
Cases (N = 67)			79.8 (5.8)				
Combined	0.941	*P* < .001					

Significance at *P* < 0.05

*AUC* Area under curve

*P value after adjustment of age and sex using univariate linear model

**Table 4:** Absence of association between RTL or LINE-1 methylation percentage and age class, sex and consanguinity

	Independent T test		Spearman correlation	
	Mean (± SD)	*P*	r	*P*
RTL				
Age class				
Children	0.96 (± 0.9)	0.88	− 0.029	0.824
Adolescents	0.91 (± 0.99)			
Sex				
Males	0.87 (± 0.9)	0.2	0.228	0.074
Females	1.2 (± 0.86)			
Consanguinity				
+ ve	1.4 (± 1.1)	0.058	0.249	0.053
− ve	0.79 (± 0.76)			
LINE-1 methylation percentage				
Age class				
Children	79.7 (± 6)	0.69	− 0.006	0.965
Adolescent	78.9 (± 6)			
Sex				
Males	79.8 (± 6)	0.45		
Females	78.3 (± 5.3)		− 0.076	0.558
Consanguinity				
+ ve	80.7 (± 5.7)	0.36	0.051	0.697
− ve	79.1 (± 6)			

Significance at *P* < 0.05

*r* correlation coefficient, + *ve* positive consanguinity, − *ve* negative consanguinity

### LINE- Methylation Level 1 as a Biomarker for Autism

Our data revealed that autistic subjects have a decreased LINE-1 methylation percentage (79.8%; ± 5.8) than the control group (88.2%; ± 3.8), *P* < 0.001 (Table 3). By applying ROC curve, LINE-1 methylation percentage is identified as a biomarker for autism: AUC = 0.889 and *P* < 0.001 (Fig. 1). By comparing LINE-1 methylation percentage between children and adolescents, as well as between males and females, no significant difference was found; *P* = 0.698 and 0.455, respectively. According to Pearson’s correlation analysis, no significant association was found between LINE-1 methylation percentage and age: r = − 0.06 and *P* = 0.96. Furthermore, the Spearman’s correlation coefficient showed that there is no significant association between LINE-1 methylation percentage and sex (r = − 0.076 and *P* = 0.558) or consanguinity (r = 0.051 and *P* = 0.69, Table 4).

## RTL and LINE-Methylation Percentage not Associated with Autism Severity

Regarding severity, there was no difference in the distribution of RTL and LINE-1 methylation percentage among mild, moderate, and severe subjects (*P* = 0.488 and 0.067, respectively). Also, no correlation was found between RTL or LINE-1 methylation percentage and severity, EEG abnormalities, GIT disturbance or sleep disorder (Table 5).

**Table 5:** Absence of association between RTL or LINE-1 methylation percentage and patient’s clinical characteristics

	Comparative means*		Spearman correlation	
	Mean (± SD)	*P*	r	*P*
RTL				
Severity				
Mild	1(± 0.9)			
Moderate	0.9 (± 0.6)	0.9	0.067	0.609
Severe	0.8 (± 1.2)			
EEG abnormalities				
+ VE	0.8 (± 0.9)	0.156	0.235	0.082
− VE	1.1 (± 0.9)			
GIT disorders				
+ VE	0.9 (± 0.9)	0.68	0.046	0.738
− VE	1 (± 1)			
Sleep disorder				
+ VE	1.1 (± 1)	0.59	− 0.080	0.556
− VE	0.9 (± 0.9)			
LINE-1 methylation percentage				
Severity				
Mild	79.6 (± 5)			
Moderate	78.1 (± 8)	0.26	− 0.173	0.190
Severe	81.9 (± 3.5)			
EEG abnormalities				
+ VE	80.6 (± 5.3)	0.17	− 0.122	0.376
− VE	78.4 (± 6.5)			
GIT disorders				
+ VE	80.1 (± 5.5)	0.23	− 0.092	0.503
− VE	78.1 (± 6.8)			
Sleep disorder				
+ VE	79.7 (± 5.7)	0.82	− 0.084	0.540
− VE	79.3 (± 6)			

*Means are compared by ANOVA in case of severity, and by independent *T* test in case of EEG abnormalities, GIT disorders and sleep disorder. Significance at *P* < 0.05

### Correlation Between RTL and LINE-1 Methylation in Autistic Subjects

We applied Pearson correlation analysis to the data of RTL and LINE-1 methylation to determine the existence of association between their decrease in autistic subjects and found that the correlation is significant (r = 0.439 and *P* < 0.001). Additionally, logistic regression analysis for the combined ROC curve of RTL and LINE-1 methylation revealed an AUC = 0.941 and a significance level of *P* < 0.001.

## Discussion

As indicated by twin studies, autism represent one of the most heritable developmental disorders and genetic factors are key players in its pathogenesis (Tick et al., 2016), however, fundamental environmental factors have been reported to have a considered implication (Hallmayer et al., 2011; Li et al., 2014). This study focused on two factors related to genetic and environmental burden. The first is altered TL which was found to increase genetic variation, involved in genomic instability, and reflect genetic predisposition (Hackett et al., 2001; Li et al., 2014), and its alteration may be attributed to genetic factors (Asghar et al., 2015; Nersisyan et al., 2019; Shubin & Greider, 2020) and environmental signals (Romano et al., 2013). Secondly, methylation percentage of the index of global methylation, LINE-1, which was reported to play a role in the neurogenesis and survival of neuronal progenitors due to its activation via the canonical WNT pathway (Kuwabara et al., 2009; Tangsuwansri et al., 2018). The involvement of both TL and LINE-1 in genomic stability, led us to explore the presence of a correlation between them in mediating autistic behaviour.

Shortening of TL induces cells to undergo senescence or apoptosis. Many causes are responsible for this shortening of telomere, including reduced telomerase expression, telomere trimming by removal of telomere loop (T-loop), and inflammation which enhance cell proliferation and cause oxidative damage to telomeric DNA and its associated proteins (Eitan et al., 2014). TL is also related to smoking, obesity and air pollution and its shortening has been linked to several neuropsychiatric disorders, such as schizophrenia, Alzheimer’s disease (Hackenhaar et al., 2021) and attention-deficit/hyperactivity disorder (Costa Dde et al., 2015). By comparing RTL between autistic patients and control subjects in our study, a significant decrease was revealed suggesting the potential role of telomere length in mediating autistic behaviour. The ability of RTL to act as biomarker for autism is suggested by the roc curve analysis with high significance *P* value and AUC. However, no correlation was found between RTL and severity of autism. The relationship between shortened TL and severe sensory symptoms was demonstrated in a previous study, which also found that cognitive functions, rather than autistic traits, are associated with TL in the parents of ASD children, however, in ASD patients, cognitive function and TL were not related (Lewis et al., 2020).

The promoter region of LINE-1 has CpG residues, these residues are regulated by Methyl-CpG binding Protein 2 (MeCP2), which has been implicated in a severe developmental disorder with autistic phenotypes and Rett syndrome (Tangsuwansri et al., 2018). Our data revealed a significant correlation between autism and a significantly decreased LINE-1 methylation percentage compared to control subjects. In addition, the high AUC determined by ROC curve analysis indicates high predictive ability of LINE-1 methylation percentage for autism. Our results differ from those of previous study which used pyrosequencing to analyze LINE-1 in DNA extracted from blood of autistic patients and found slight non-significant decrease in LINE-1 methylation (Garcia-Ortiz et al., 2021). The variance in outcome may be attributable to the precise positions of CpG sites analyzed or the method of analysis. Previous research utilized lymphoblastoid cell lines demonstrated that INE-1-inserted differentially expressed genes are associated with ASD-related mechanisms, such as sex hormone receptor signalling and axon guidance signalling. LINE-1 methylation was also found to be reduced in cell lines derived from patients with severe language impairment (Tangsuwansri et al., 2018). In our study, no association was observed between of LINE-1 methylation and severity, EEG abnormalities, or GI disorders.

LINE-1 hypomethylation is suggested to decrease the methylation of sub-telomeric regions, which are associated with short TL (Wong et al., 2014). In 2017, a study on healthy adolescent showed an association between lower levels of global DNA methylation and shorter TL which may decrease genome stability and raise the susceptibility to diseases (Dong et al., 2017). Considering that both RTL and LINE-1 methylation contribute to vulnerability of autism, we applied Pearson correlation to examine the association between RTL and LINE-1 methylation in autistic patients and found that this association is confirmed and statistically significant. Moreover, logistic regression analysis revealed an increase in the predictive efficacy of the combined biomarkers for autism as indicated by the increase in AUC value. These findings suggest the involvement of LINE-1 hypomethylation and shorter TL in the decreased genomic stability in autistic individuals (Williams et al., 2013) and the ability of using them as predictive biomarkers for autism. Additionally, these results support the previous findings which suggest that LINE-1 hypomethylation is linked to telomere shortening, whereas DNA methylation is one of the mechanisms that control TL and maintenance, and telomere dysfunction can cause LINE-1 retrotransposition (Chang et al., 2015).

Altogether, the study has concluded that RTL and LINE-1 methylation percentage are reduced in autism without affecting its severity. However, the study has been carried out on a small number of subjects, thus, in the future research we plan to increase the number of subjects and analyze the expression of the two open reading frames (ORF1 and ORF2) of LINE-1 and genes involved in regulating the methylation machinery in autism.
